# Increased *Ifng* and *Il10* Expression Correlate with Disease in Rodent Models Experimentally Infected with Modoc Virus

**DOI:** 10.3390/v14051026

**Published:** 2022-05-11

**Authors:** Tyler J. Sherman, Douglas Petty, Tony Schountz, Natasha Hodges, Ann C. Hawkinson

**Affiliations:** 1 Department of Microbiology, Immunology, and Pathology, College of Veterinary Medicine, Colorado State University, Fort Collins, CO 80523, USA; douglas.petty@colostate.edu (D.P.); tony.schountz@colostate.edu (T.S.); natasha.hodges@colostate.edu (N.H.); 2 School of Biological Sciences, College of Natural and Health Sciences, University of Northern Colorado, Greeley, CO 80524, USA; ahawkinson2@coloradomtn.edu

**Keywords:** flavivirus, *Peromyscus*, *Mesocricetus*, interferon-gamma, interleukin-10, gene expression, rodent-borne virus

## Abstract

Flaviviruses present an ongoing threat to global public health, although the factors that contribute to the disease remain incompletely understood. We examined an acute Modoc virus (MODV) infection of two rodent models. Viral RNA was detected in the kidneys, spleen, liver, brain, urine, and sera of experimentally infected deer mice, a reservoir host of MODV, and Syrian hamsters, a known disease model. As expected, clinical outcomes differed between species, and the levels of viral RNA recovered from various tissues demonstrated signs of differential replication and tissue tropism. Multivariate analysis indicated significance in the profile of expressed genes between species when analyzed across tissues and over time (*p* = 0.02). Between-subject effects with corrected models revealed a significance specific to the expression of *Ifng* (*p* = 0.01). the expression of *Ifng* was elevated in hamsters as compared to deer mice in brain tissues at all timepoints. As the over-expression of *Ifng* has been shown to correlate with decreased vascular integrity, the findings presented here offer a potential mechanism for viral dissemination into the CNS. The expression of *IL10* also differed significantly between species at certain timepoints in brain tissues; however, it is uncertain how increased expression of this cytokine may influence the outcome of MODV-induced pathology.

## 1. Introduction

Modoc virus (MODV) is a rodent-borne flavivirus isolated from deer mice (*Peromyscus maniculatus*) in North America [1]. As with all members of the *Flavivirus* genus, MODV is enveloped and contains a positive-sense RNA genome that encodes three structural (envelope, E; capsid, C; pre-membrane/membrane, prM) and seven non-structural (NS1, NS2A, NS2B, NS3, NS4A, NS4B, NS5) proteins. A 5′ methyl cap protects the viral genome from host cell recognition and provides ribosomal localization, while non-coding elements at the 3′ end contribute to viral infectivity and transmission, replication, and host pathogenesis [2,3,4]. Unlike most medically relevant flaviviruses, which have arthropod vectors, MODV was exclusively isolated from *Peromyscus* spp. in nature and fails to replicate in invertebrate cell lines [1,5,6,7]. Thus, MODV belongs to a distinct cluster of flaviviruses with no known vector (NKV) [8]. To date, only a few instances of human disease caused by NKV flaviviruses have been documented [9,10]. Nevertheless, MODV may provide an alternative model for flavivirus research, because it can be manipulated at biosafety level-2 (BSL-2) and can recapitulate human-like flavivirus disease in Syrian golden hamsters (*Mesocricetus auratus*) [11,12].

Flaviviruses are globally distributed and can elicit a range of clinical pathologies. Although most human infections are subclinical and self-resolving, instances of severe disease may result in multiple-organ failures, autoimmune disorders, malformations in fetal development, hemorrhagic fevers, or meningoencephalitis with permanent neurologic sequelae [13,14]. The increasing spread of vector-borne flaviviruses in recent decades has evoked renewed concern for the burden of these viruses on global public health [15,16]. For example, dengue virus (DENV) is believed to infect more than 390 m individuals annually, leading to clinical disease in approximately 96 million [8,17]. Likewise, West Nile virus (WNV), Japanese encephalitis virus (JEV), yellow fever virus (YFV), and Zika virus (ZIKV) continue to threaten individuals worldwide, with a conservative estimate of annual infections approaching 4 billion [18,19,20,21,22]. The issue of exposure is further compounded by ongoing climate variability, which may extend the realized niche for vectors such as ticks and mosquitoes, which carry and transmit flaviviruses [23,24,25,26]. Combined with the relative lack of flavivirus vaccines and FDA-approved antiviral therapies, there is a need for continued research [17].

As a confirmed and putative reservoir host of several human pathogens, deer mice offer a unique model system for examining the immune processes that control infection while preventing disease [27]. In contrast, Syrian golden hamsters are frequently used as models that recapitulate human-like disease from many pathogens without the need for genetic manipulation or chemical immunocompromise that is necessary in mouse models [28,29]. Indeed, both models have been used to study facets of MODV ecology and infection and have revealed clues regarding the tropism and subsequent pathology of MODV in vivo [5,12,30,31,32,33]. For example, MODV can induce a poliomyelitis-like syndrome in hamsters but not in deer mice that are experimentally infected through the same route [11,32]. Despite this, no studies have attempted to examine the presence of differentially expressed immune genes during MODV infection in these animals to date. Therefore, this research used MODV to directly compare viral replication kinetics, tissue tropism, and immune gene expression between deer mice and hamsters during acute experimental infection. It was discovered that MODV RNA could be recovered in greater amounts from the liver and spleen of deer mice. In contrast, higher levels of viral RNA were detected in the brain, sera, urine, and kidneys of hamsters, particularly beginning at day 4 post-infection. Examining the gene expression of five important immune cytokines (*Ifng*, *Tnf*, *Il6*, *Il10* and *Tgfb*) revealed notable variability in the detectable levels of these transcripts in each model species. Although these cytokines represent only a small subset of the signals involved in an antiviral response, they were selected for their ability to coordinate important facets of the immune system and for their previous implication in various flavivirus-induced pathologies [34,35,36]. Additionally, the IFN-stimulated gene (ISG) *Oas1b* was chosen as a proxy to gauge the type I interferon (IFN-α/β) response to MODV. Despite the ability of many flaviviruses to antagonize IFN signaling; it was found that both rodents were able to express transcripts of *Oas1b*, albeit at significantly different levels in certain tissues and at different timepoints.

## 2. Materials and Methods

### 2.1. Experimental Infections

Deer mice were supplied from an established colony at the University of Northern Colorado, whereas Syrian hamsters were purchased from Envigo Laboratories. In total, 36 rodents (18 of each species, 50/50 mixed sexes), ranging from 10 to 16 weeks of age, were intraperitoneally inoculated with 0.3 mL of MODV M544 (ATCC, Manassas, VA, USA; passaged once in Vero E6 cells) at an infectious dose of ~10^4.2^ TCID_50_. Similarly, 12 negative controls (6 of each species, 50/50 mixed sexes) were intraperitoneally sham inoculated with 0.3 mL of 1× DPBS (pH = 7.3). Following inoculation, all animals were housed individually, monitored daily, and euthanized immediately following the development of signs of morbidity (not including minor changes in behavior (e.g., lethargy) or weight loss). Animals challenged with virus were serially euthanized in groups of three on days 1, 2, 4, 6, 8, and 10 post-infection (p.i.). Negative controls were euthanized in groups of two on days 1, 6, and 10 p.i. Necropsies were performed immediately following euthanasia and included the collection of urine (extracted directly from the bladder), blood sera (collected via cardiac exsanguination), kidneys, liver, spleen, and brain. All collected tissues were frozen immediately in liquid nitrogen baths and stored at −80 °C until further use.

### 2.2. Viral RNA Extraction and RT-qPCR

Organ samples were prepared for viral RNA extraction by first homogenizing 30 mg of tissue with zirconia/silica beads in 600 µL of sterile 1× PBS. Homogenates were centrifuged through a QiaShredder column (Qiagen, Germantown, MD, USA) at 14,000× *g* for 8 min at 4 °C. A total of 140 µL of tissue supernatant, urine, or blood sera were then used for extraction using a QIAmp Viral RNA Mini kit (Qiagen) according to the manufacturer’s protocol. To reverse-transcribe viral RNA, 2 µL of sample were mixed with 5.2 µL of nuclease-free water and 2.8 µL of MODV-specific primers at a final concentration of 280 nM (Table 1). RNA and primers were incubated at 94 °C for 1 min and then placed in an ice bath for 3 min before the addition of 0.5 µL Episcript RT enzyme (Lucigen, Radnor, PA, USA), 2 µL 10 × RT buffer, 2 µL of DTT, 1 µL dNTPs at 10 mM, 0.5 µL RNase inhibitor (New England BioLabs, Ipswich, MA, USA), and 4 µL nuclease-free water. First-strand cDNA synthesis was performed by incubating the reaction mixture at 45 °C for 1.5 h followed by 5 min at 85 °C to inactivate the RT.

Viral cDNA from collected tissues was examined using RT-qPCR and the same MODV-specific primers referenced above. In brief, 2 µL of cDNA were combined with 10 µL of PerfeCTa SYBR FastMix (Quantabio, Beverly, MA, USA), 2 µL of primers (each at a working concentration of 3 µM), and 6 µL of nuclease-free water. Cycling was conducted on a CFX384 Touch (BioRad, Hercules, CA, USA) with the following conditions: initial denaturation at 95 °C for 10 min followed by 40 cycles at 95 °C for 15 s and 60 °C for 1 min. To compare relative quantities of recovered virus, 1:10 serial dilutions of stock MODV cDNA (prepared using the same protocol as above) were used to generate four standard curves, from which the means were used to represent the tissue culture infective dose 50 (TCID_50_) “equivalent titer” of recovered viral RNA.

### 2.3. Cellular RNA Extraction and Gene Expression Analysis

Tissues collected from MODV-infected and control animals were used to examine the relative expression of select immune genes on days 1, 6, and 10 p.i. In brief, 30 mg of tissue from each organ of interest (kidney, liver, spleen and brain) were first homogenized using zirconia/silica beads in a solution of TRK lysis buffer from an E.Z.N.A Total RNA kit (Omega Bio-Tek, Norcross, GA, USA) with the addition of 20 µL/mL of β-mercaptoethanol. Suspensions were then transferred to a QiaShredder column and centrifuged at 14,000× *g* for 8 min at 4 °C to further mechanically lyse cells and pellet cellular debris. Supernatants were carefully removed and subjected to RNA extraction using the above kit according to the manufacturer’s protocol. Prior to cDNA synthesis, all samples were treated to remove genomic DNA using a PerfeCTa DNase I kit (Quantabio, Beverly, MA, USA) according to the manufacturer’s protocol. First-strand cDNA was synthesized by mixing the entire DNase-treated reaction with 4 µL of qScript cDNA SuperMix (Quantabio) and 5 µL of nuclease-free water, followed by incubation at 25 °C for 10 min, 42 °C for 30 min, and then 85 °C for 5 min.

Analysis of relative immune gene expression was conducted by combining 2 µL of template cDNA (diluted to 10 ng/µL) with 5 µL of SsoAdvanced SYBR Supermix (BioRad), 2 µL of forward and reverse primers, each at 2 µM, and 1 µL of nuclease-free water. All primers (Table 1 and Table 2) were used at a final concentration of 200 nM except for hamster primers *Il6* and *Ifng*, which were used at final concentrations of 400 nM and 100 nM, respectively. Primers for the *Oas1b* gene were designed using homologous sequences found in regions of the *Oas1b* coding transcripts from deer mice, golden hamsters, and laboratory house mouse (*Mus musculus*). To determine primer efficiencies, standard curves were first generated using the mean Ct values from duplicate runs of samples in a 1:10 dilution series. The slope values from these curves were then used to determine efficiency using the following equation: efficiency (%) = (10^(−1/slope value)−1) × 100.

All samples were run in duplicate on a CFX384 Touch thermocycler with the following cycle conditions: initial denaturation at 95 °C for 10 min followed by 40 cycles at 95 °C for 15 s and 60 °C for 1 min. Melt curve analyses were included with each assay to verify single product amplification. Additionally, a no-template control (NTC) was included with each assay. Relative gene expression was calculated with mean Ct values using the comparative Ct method (i.e., ΔΔCt) as proposed by Livak and Schmittgen, whereby immune genes were first normalized against the constitutively expressed β-actin gene (ΔCt) and then calibrated against the ΔCt of mock-infected controls (ΔΔCt) [40]. Finally, relative gene expression was depicted as the mean fold change (2^−ΔΔCt^) ± standard error of the mean (SEM) between infected and mock-infected animals at each of the previously described collection timepoints. Samples that did not generate a Ct value were given a default value of 40.

**Table 2:** Sequence data and amplification efficiency for *Mesocricetus auratus* primers.

Target		Sequence (5′ to 3′)	bp	R^2^	Eff. (%)	Accession	Reference
*β-Actin*	F R	GCTACAGCTTCACCACCACA TCTCCAGGGAGGAAGAGGAT	123	1.000	102.4	XM_013120404.2	[37]
*Oas1b*	F R	CAGTATGCCCTGGAGCTGC GTACTTGGTGACCAGTTCC	111	0.999	103.5	XM_013119795.2	This study
*Ifng*	F R	TGTTGCTCTGCCTCACTCAGG AAGACGAGGTCCCCTCCATTC	130	1.000	104.2	AF034482.1	[41]
*Tnf*	F R	TGAGCCATCGTGCCAATG AGCCCGTCTGCTGGTATCAC	79	0.998	97.7	XM_005086799.3	[41]
*Tgfb*	F R	TGTGTGCGGCAGCTGTACA TGGGCTCGTGAATCCACTTC	63	1.000	100.7	XM_013125593.2	[29]
*IL6*	F R	CCTGAAAGCACTTGAAGAATTCC GGTATGCTAAGGCACAGCACACT	78	1.000	112.4	XM_005087110.2	[29]
*IL10*	F R	GGTTGCCAAACCTTATCAGAAATG TTCACCTGTTCCACAGCCTTG	194	1.000	99.5	XM_021232886.1	[41]
*MODV-NS5*	F R	CCAGGACAAGTCATGTGGTAGC TCCCAAAGATGTTCCTCACCTT	101	0.998	107.5	NC_003635.1	[39]

### 2.4. Statistical Analysis

A log_10_ transformation was first applied to all relative gene expression values prior to any downstream statistical analysis. Following transformations, SPSS v25.0 (IBM, Armonk, NY, USA) was used to conduct a MANOVA to determine significant differences in the expression values of immune genes between species among select tissues at distinct collection timepoints. Additionally, separate MANOVA tests were conducted for each species to examine differences in gene expression profiles within groups when tissues and collection timepoints were held as fixed factors. Independent samples’ *t*-tests were conducted using PRISM v8.3.1 (GraphPad, La Jolla, CA, USA) to determine whether the expression of genes differed significantly between species within a particular tissue and specific collection timepoint. Finally, Mann–Whitney tests were conducted in PRISM to determine whether viral loads differed significantly between species in particular tissues over time. For all analyses, significant differences between groups were evaluated with α = 0.05.

## 3. Results

### 3.1. Variable Clinical Outcomes of MODV Infection

Both deer mice and hamsters are susceptible to MODV; however, the clinical outcomes of acute infection differ considerably. Over the 10-day experimental period, deer mice exhibited no overt signs of disease (e.g., ruffled fur or piloerection, fluid discharge from the nose, mouth or eyes, hematuria, muscle weakness or tremors, weight loss, or other peculiar changes in behavior). Likewise, there were no notable indications of gross organ pathology upon necropsy of any of the infected individuals. 

**Figure 1:**
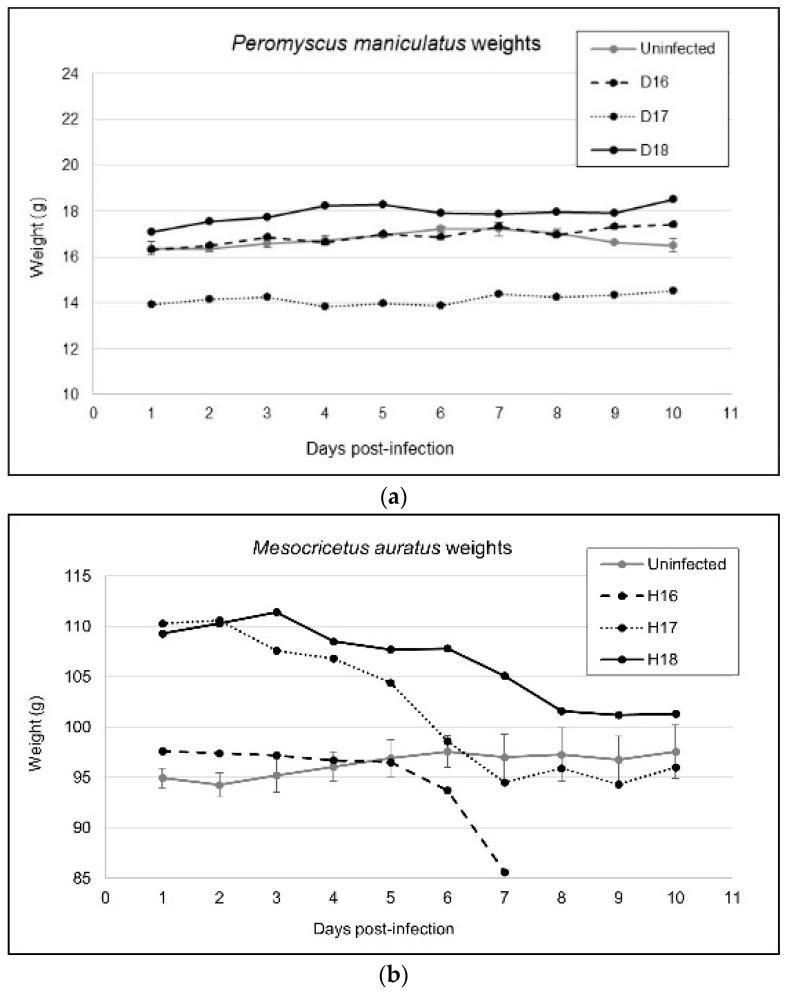
Weight gain or loss in (**a**) *Peromyscus maniculatus* and (**b**) *Mesocricetus auratus* over the experimental period. Each datapoint represents the weight for a particular infected individual at that timepoint or the mean (+/− SEM) weight of two mock-infected individuals at the same timepoint.

In contrast, hamsters began to exhibit lethargy and weight loss as early as day 2 p.i. (Figure 1). Gross organ pathologies became apparent beginning day 4 p.i., with nearly all infected individuals demonstrating splenomegaly and mild to moderate thoracic hemorrhage at the time of necropsy. Past this timepoint, all males presented with severe epididymitis and orchitis upon necropsy. On day 7, one female (H16) was euthanized early due to apparent limb paralysis, tachypnea, nasal hemorrhage, and incontinence. Two more hamsters developed similar signs of disease with neurologic involvement by day 8 p.i. and were euthanized immediately (which coincided with the predetermine necropsy schedule). By day 10 p.i., the remaining hamsters demonstrated extreme lethargy, slow/weak reflexes in the front limbs, splenomegaly, orchitis, and extreme weight loss. In total, 12/18 (67%) infected hamsters demonstrated some form of gross anatomical pathology or clinical manifestation of disease within the 10-day experimental period. None of the uninfected controls from either rodent species presented with signs of illness throughout the experimental period.

### 3.2. Viral Replication Kinetics

**Figure 2:**
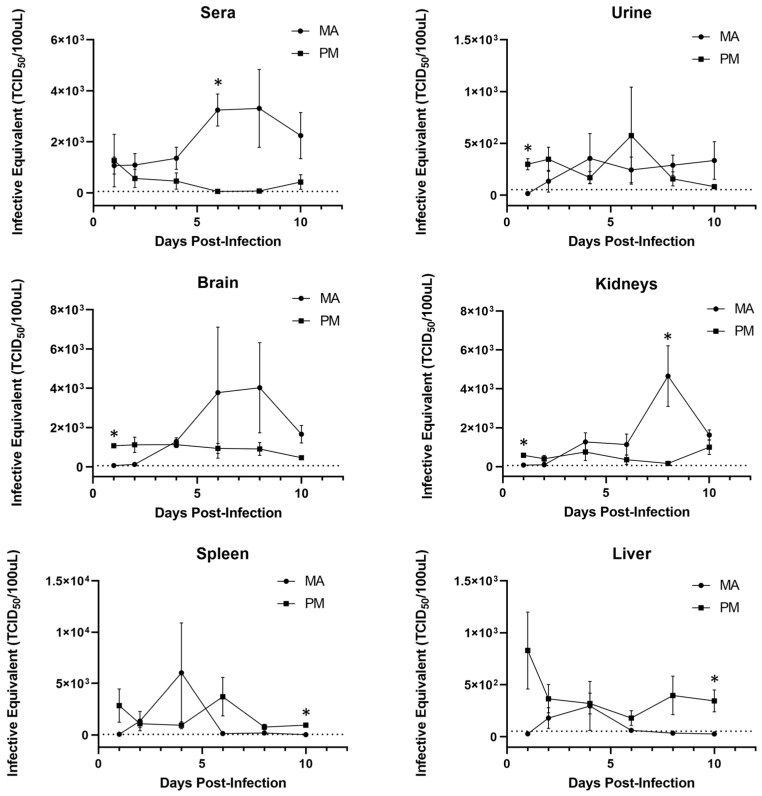
Viral RNA detected in select tissues and fluids of *Peromyscus maniculatus* (PM, squares) and *Mesocricetus auratus* (MA, circles). Viral RNA quantified via RT-qPCR was translated into TCID_50_ equivalent titers. Datapoints represent the mean (+/− SEM) viral load recovered from infected replicates at each timepoint (*n* = 3). Asterisks (*) represent a significant difference in RNA loads between each species at a particular timepoint, as determined by Mann–Whitney tests. Dotted lines represent the limit of detection at an infective equivalent of approximately 10^1.7^ TCID_50_.

To assess differences in viral replication kinetics between deer mice and hamsters, RT-qPCR was used to quantify the levels of MODV RNA expressed in select tissues and fluids over time (Figure 2). It was determined that MODV RNA is generally recovered at higher levels in the spleens and livers of deer mice as compared to the urine, sera, brains, and kidneys of hamsters during an acute infection period. Interestingly, in all examined tissues and fluids, MODV RNA levels began to increase by day 4 p.i. in hamsters, and decreased at the same timepoint in deer mice (except in spleens and livers, where a transient increase in viral RNA occurred on days 4 and 6, respectively). Despite these observed trends, the variance in recovered viral RNA between individuals, especially hamsters, was quite high. This finding may be reflective of minor genetic nuances between individuals and support the need for additional replicates at each timepoint in future studies.

### 3.3. Differential Transcript Expression

The differential expression of *Ifng*, *Tnf*, *Il6*, *Il10*, and *Tgfb* during acute MODV infection in both hamsters and deer mice was examined using RT-qPCR. Following the transformation of relative gene expression values, a Shapiro–Wilk test for normality confirmed that all values were normally distributed in deer mice (*p* ≥ 0.11), except for one instance (*Tgfb* in liver tissues; *p* = 0.04). Similarly, all values in hamsters were found to be normally distributed (*p* ≥ 0.09) except for *Ifng* expression in the spleen (*p* = 0.02) and brain (*p* = 0.21) as well as the expression of *Il10* in the brain (*p* = 0.04). To determine whether the observed levels of transcript expression were statistically meaningful, multivariate analysis of variance (MANOVA) models were used to examine transcript expression profiles in each species, first separately and then together. A significant difference in the complete profile of cytokine transcripts expressed between tissues over time was found for each species (deer mice, *p* = 0.01; hamsters, *p* = 0.01). In deer mice, between-subjects effects with corrected models revealed significant differential expression of *Ifng*, *Tnf*, and *Tgfb* when both tissue and collection timepoint were treated as cofactors. In hamsters, *Ifng*, *Tnf*, *Il6*, and *Il10* were found to exhibit significant differential expression, but *Tgfb* was not. When a single MANOVA compared the profiles of transcript expression between species, collection timepoints, and tissues, a significant difference was also found in the relative expression of all transcripts over time between tissues and species (*p* = 0.02). In this analysis, only the expression of *Ifng* differed significantly (*p* = 0.01). Despite the trends observed regarding expression of *Il10* in brain tissues, levels of *Il10* in this study are significant when only tissue and species or tissue and timepoint of collection are treated as fixed factors in multivariate analysis (*p* ≤ 0.01). Nevertheless, these findings confirm that infection by MODV elicits a unique immune response in each rodent model when the titer and route of viral challenge is standardized. In line with this, there is preliminary support of the idea that the regional cytokine milieu may influence the outcome of both local and systemic disease pathology or resistance.

**Figure 3:**
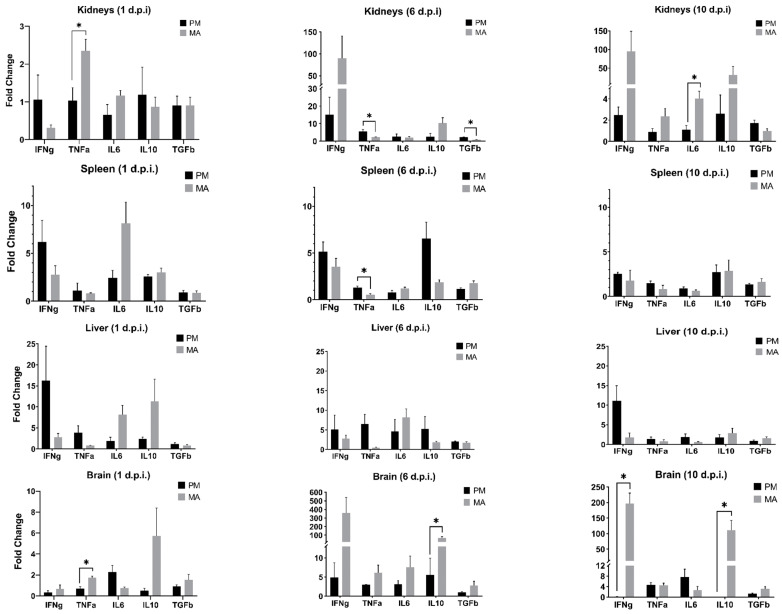
Cytokine gene expression levels as detected by RT-qPCR in select tissues from *Peromyscus maniculatus* (PM) and *Mesocricetus auratus* (MA). Each timepoint reflects the mean relative expression levels from infected replicates (*n* = 3) of each species. Bars with asterisks (*) denote a significant difference in the expression levels between species at that collection timepoint (uncorrected for multiple tests).

Independent samples’ *t*-tests were used to compare the mean level of transcript expression between species in specific tissues at specific timepoints (Figure 3). The patterns of expression between tissues generally corresponded with the detected MODV levels. For example, *Ifng* expression was initially higher for deer mice in the spleen and liver, both of which contained marginally higher levels of viral RNA. Similarly, the expression of *Ifng* in the kidneys (past day 1) and brains of infected hamsters mirrored elevated levels of viral RNA recovered from those tissues. Indeed, one would expect to recover antiviral transcripts such as *Ifng* concomitant with higher viral loads; however, these trends may be tissue-dependent, as suggested by the examination of one particular hamster, which demonstrated much higher levels of both viral RNA and *Ifng* on day 6 in the kidneys but only high levels of viral RNA (and the lowest expression of *Ifng* among all bio-replicates) in the brain at this same timepoint. In some instances, transcript expression significantly differed between species; however, these cases only occurred in certain tissues and at specific timepoints.

### 3.4. Oas1b Expression

**Figure 4:**
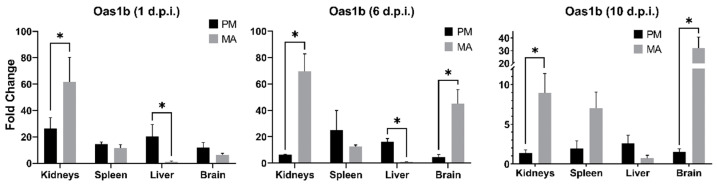
Expression levels of *Oas1b* in *Peromyscus maniculatus* (PM) and *Mesocricetus auratus* (MA) tissues as detected by RT-qPCR. Each timepoint reflects the mean relative expression levels from infected replicates (*n* = 3) of each species. Bars with asterisks (*) denote a significant difference in relative expression between species (uncorrected for multiple tests).

RT-qPCR was additionally used to examine the expression levels of the interferon-stimulated gene (ISG) *Oas1b*. Using a three-factor analysis of variance (ANOVA), it was discovered that expression of *Oas1b* significantly differed between deer mice and hamsters in select tissues at distinct timepoints (*p* = 0.03). Namely, expression in hamster kidneys at all timepoints, as well as in brain tissues at days 6 and 10 p.i., was much higher than those of deer mice at the same timepoints (Figure 4). In contrast, expression in deer mouse livers was significantly higher on days 1 and 6, but not on day 10 p.i.

## 4. Discussion

This study used a comparative design to investigate MODV replication kinetics, tissue tropism, and cytokine response in contrasting rodent models. Deer mice, reservoir hosts of MODV, showed no signs of disease during experimental infection. This corroborates previous research and confirms that, even in a higher-titer challenge, deer mice can resist disease [31,32]. In contrast, over two-thirds of infected hamsters exhibited disease reminiscent of past studies [11,12]. Adams et al. describe an experiment in which hamsters developed bilateral hindlimb paralysis by day 6 p.i. [12]. Leyssen et al. discovered that MODV-infected hamsters develop a similar neurologic disease to encephalitic flaviviruses (e.g., WNV or JEV), by day 10 p.i. [11]. These findings all support the use of hamsters and deer mice as comparative models and may provide the impetus to further explore the differential expression of immune genes to better understand mechanisms of disease resistance.

Regarding tissue tropism, past investigations suggest that MODV differentially replicates in the spleen, lungs, and salivary-submaxillary glands of deer mice, and in the kidneys and brain of hamsters [11,12,30,31,32,39]. The results presented here broadly corroborate previous works and further demonstrate the relatively higher recovery of viral RNA from the livers and sera of deer mice and hamsters, respectively. More specifically, sera levels were similar at the first sampling timepoint but began to diverge by day 4 post-infection. By day 6, peak levels of viral RNA were reached in hamster sera, which differed significantly from those found in deer mice. Taken together, these findings may offer clues concerning the pathogenic progression of MODV. For example, increased viremia in hamsters may better facilitate viral dissemination into the central nervous system (CNS). It has been demonstrated that other flaviviruses may infiltrate the CNS via cytokine-mediated breakdown of the blood–brain barrier (BBB) or via the translocation of viral particles into the brain parenchyma across the infected epithelium [42]. However, it is important to acknowledge that any observed levels of viral RNA do not necessarily imply the presence of infectious virus; therefore, further investigation will be required.

For this study, a small subset of antiviral genes was chosen for examination based on their previous implication during flavivirus infection [34,43,44,45]. Of particular interest, we found that transcripts of *Ifng* in the brains of infected hamsters were elevated above those in deer mice on all days sampled. Extreme differences were observed by day 6 and were significant by day 10 p.i. These trends were surprising to observe given that levels of viral RNA were initially higher in deer mouse brain tissues. *Ifng* is a multifunctional, pleiotropic cytokine critical for the control of pathogens [46]. It has been cited as one of the most potent activators of classical macrophages, induces nitric oxide production, and stimulates the upregulation of MHC class I and II, each of which aid in the response against viruses [46]. Despite its antiviral role, some research suggests that over- or under-expression may lead to deleterious clinical outcomes [47]. For example, intact *Ifng* signaling is required to promote the protective roles of innate γδ T cells and primary dendritic cells against WNV infection [48]. Nevertheless, the extreme upregulation of *Ifng* may lead to the overexpression of intercellular adhesion molecule-1 (ICAM-1), a putative binding receptor for many flaviviruses, including WNV [49].

Unlike *Ifng*, interleukin-10 (*Il10*) is canonically regarded as immunomodulatory or suppressive [50]. Although the exact mechanisms of its function are not fully understood, *Il10* may mollify inflammation by interfering with the production of pro-inflammatory cytokines or chemokines concomitant with the downregulation of MHC II expression [50]. It is, therefore, curious that the expression of *Il10* transcripts in the brain of infected hamsters far exceeds those in deer mice at all timepoints sampled. Indeed, previous research has correlated *Il10* with both protective and adverse outcomes of flavivirus infection [51]. These dichotomous outcomes may be serotype-specific, as some research has shown *Il10* to be elevated during infection with DENV-2 while demonstrating active downregulation in response to DENV-1, 3, and 4 [52]. Moreover, multiple studies have suggested that the excessive production of *Il10* could inhibit the protective effects of T cells [53,54,55]. Further investigation will be required to elucidate the role of *Il10* in this particular model system; however, it is interesting to find that high expression levels are correlated with neuropathologic outcomes in hamsters.

In addition to shaping the cytokine profile of the immune response, many flaviviruses antagonize type I IFN (IFN-α/β) signaling at multiple levels and may subsequently inhibit the expression of intracellular, antiviral effector proteins [56,57]. Due to the paucity of available sequences for deer mouse and hamster type I IFN transcripts, we chose to examine the expression of *Oas1b* as a representative IFN-stimulated gene (ISG). Canonically, Oas (oligoadenylate-synthetase) proteins exert their antiviral effect by activating latent cytosolic ribonuclease L (RNAse L), an enzyme that acts to degrade viral transcripts. Although *Oas1b* lacks this enzymatic activity, and therefore exerts antiviral activity through other, incompletely understood mechanisms, it has nevertheless been demonstrated to be protective against some flavivirus infections [58,59,60,61]. It was interesting to find that significantly higher expression of *Oas1b* occurred in the livers of infected deer mice as opposed to the kidneys and brains of infected hamsters (Figure 4). These patterns roughly coincide with the apparent tropism of MODV toward these tissues, as determined by the quantities of viral transcripts recovered from each species; however, the same patterns of expression were not observed in the spleens of infected animals. Given these outcomes, MODV does not appear to antagonize the ISG response in a manner characteristic of some other flaviviruses [62]. Moreover, the high expression of *Oas1b* by 24 h p.i. confirms that both species are capable of recognizing MODV molecular patterns and initiating the expression of ISGs within a comparable timeframe to other well-studied flaviviruses [63].

It is unclear how the patterns of *Tnf*, *Il6*, and *Tgfb* influence MODV replication in either deer mouse or hamster tissues. For example, expression of *Tnf* differed significantly between deer mice and hamsters on day 1 in the kidneys and brain, as well as day 6 in the kidneys and spleen, but at no other timepoints in any tissues. The expression of *IL6* differed significantly only at day 10 in the kidneys while *Tgfb* differed only at day 6 in the kidneys. On one hand, the relative lack of variable expression of these three cytokines suggests negligible roles under the parameters of this experiment; however, the occasional instances of statistical significance may warrant further investigation. Future studies should include an immunohistopathologic inspection of infected tissues, comprehensive transcriptomic profiles, and assays that quantify translational end products of the cytokines examined [50]. In addition, increasing cohort sample sizes would likely help to further elucidate the observed trends outlined in the work presented here.

In conclusion, this study contributes to a growing body of knowledge examining the contribution of select immune genes in correlation with diverse clinical outcomes of viral pathogenesis. Specifically, we highlight the potential importance of two cytokines, *Ifng* and *Il10*, in the development of flavivirus-induced neurologic disease.

## Figures and Tables

**Table 1:** Sequence data and amplification efficiency for *Peromyscus maniculatus* primers.

Target		Sequence (5′ to 3′)	bp	R^2^	Eff. (%)	Accession	Reference
*β-Actin*	F R	GCTACAGCTTCACCACCACA TCTCCAGGGAGGAAGAGGAT	123	1.000	102.0	XM_006998174.2	[37]
*Oas1b*	F R	CAGTATGCCCTGGAGCTGC GTACTTGGTGACCAGTTCC	111	0.998	98.3	XM_006970848.1	This study
*Ifng*	F R	ACAGCAGTGAGGAGAAACGG GACAGGCGGTACATCACTCC	115	0.970	94.7	AY289494.1	This study
*Tnf*	F R	GGGCTGTACCTCGTCTACTC ACAGGAGGTTGACTTTGTCC	121	0.999	100.4	XM_006995235.2	This study
*Tgfb*	F R	CGTGGAACTCTACCAGAAATACAGC TCAAAAGACAACCACTCAGGCG	96	0.999	95.6	XM_006988036.2	[38]
*IL6*	F R	CCATCCAACTCATCCTGAAAGC CCACAGATTGGTACACATAGGCAC	101	0.999	96.1	AY256518.1	[38]
*IL10*	F R	CAGACCTACACGCTTCGAG CCCAGGTAACCCTTAAAGTCC	128	0.999	111.2	XM_006995328.2	This study
*MODV-NS5*	F R	CCAGGACAAGTCATGTGGTAGC TCCCAAAGATGTTCCTCACCTT	101	0.998	107.5	NC_003635.1	[39]
